# Antibacterial Properties and Efficacy of a Novel SPLUNC1-Derived Antimicrobial Peptide, α4-Short, in a Murine Model of Respiratory Infection

**DOI:** 10.1128/mBio.00226-19

**Published:** 2019-04-09

**Authors:** Shasha Jiang, Berthony Deslouches, Chen Chen, Matthew E. Di, Y. Peter Di

**Affiliations:** a Department of Environmental and Occupational Health, Graduate School of Public Health, University of Pittsburgh, Pittsburgh, Pennsylvania, USA; UC Berkeley

**Keywords:** SPLUNC1, airway infection, antibiotic resistance, antimicrobial peptides, biofilm, broad spectrum, drug resistance, *in vivo* efficacy, multidrug-resistant bacteria, peptide antibiotics, pneumonia, selective toxicity

## Abstract

Multidrug resistance (MDR) by bacterial pathogens constitutes a global health crisis, and resistance to treatment displayed by biofilm-associated infections (e.g., cystic fibrosis, surgical sites, and medical implants) only exacerbates a problem that is already difficult to overcome. Antimicrobial peptides (AMPs) are a promising class of therapeutics that may be useful in the battle against antibiotic resistance, although certain limitations have hindered their clinical development. The goal of this study was to examine the therapeutic potential of novel AMPs derived from the multifunctional respiratory host defense protein SPLUNC1. Using standard growth inhibition and antibiofilm assays, we demonstrated that a novel structurally optimized AMP, α4-short, was highly effective against the most common group of MDR bacteria while showing broad-spectrum bactericidal and antibiofilm activities. With negligible hemolysis and toxicity to white blood cells, the new peptide also demonstrated *in vivo* efficacy when delivered directly into the airway in a murine model of Pseudomonas aeruginosa-induced respiratory infection. The data warrant further exploration of SPLUNC1-derived AMPs with optimized structures to assess the potential application to difficult-to-cure biofilm-associated infections.

## Abstract (precis)

The rise of superbugs underscores the urgent need for novel antimicrobial agents. Antimicrobial peptides (AMPs) have the ability to kill superbugs regardless of resistance to traditional antibiotics. However, AMPs often display a lack of efficacy *in vivo.* Sequence optimization and engineering are promising but may result in increased host toxicity. We report here the optimization of a novel AMP (α4-short) derived from the multifunctional respiratory protein SPLUNC1. The AMP α4-short demonstrated broad-spectrum activity against superbugs as well as *in vivo* efficacy in the P. aeruginosa pneumonia model. Further exploration for clinical development is warranted.

## INTRODUCTION

The prevalence of multidrug-resistant (MDR) bacteria constitutes a major health crisis. Bacteria have the ability to display multiple mechanisms of resistance. In addition, they have the ability to develop a biofilm mode of growth, which is associated with inherently enhanced resistance to treatment and, thus, often difficult to eradicate. ESKAPE pathogens (Enterococcus faecium, Staphylococcus aureus, Klebsiella pneumoniae, Acinetobacter baumannii, Pseudomonas aeruginosa, and *Enterobacter* species), are considered the most common group of MDR bacteria (1–4). Many of these organisms are frequently responsible for nosocomial infections and contribute to high rates of morbidity and mortality (e.g., ventilator-associated pneumonia) (4–7). Moreover, some ESKAPE pathogens may also cause severe community-acquired diseases such as S. aureus-induced foot infection in diabetic patients (8) or chronic lung infection in cystic fibrosis (CF) patients due to P. aeruginosa colonization (9). ESKAPE pathogens also share the ability to form biofilm, which may render ESKAPE infections difficult to treat (10). In the last few decades, antimicrobial peptides (AMPs) have emerged as potential therapeutics against these MDR pathogens due to their ability to perturb bacterial membranes by interactions with negatively charged phospholipids on the surfaces of these cells. Several studies have shown that bacteria are less able to elicit drug resistance against AMPs compared to standard antibiotics (3, 11, 12). However, the lack of evidence for *in vivo* efficacy in animal models of infection has hindered the clinical development of AMPs.

We previously reported the antibiofilm activity of an AMP derived from the human respiratory host defense protein SPLUNC1 (short palate lung and nasal epithelial clone 1) (13–16). This protein is 256 amino acid residues long and is an important component of innate immunity (16–19). In addition, it acts as a fluid-spreading surfactant, which facilitates mucociliary clearance of bacteria and foreign particles or particulates such as nanoparticles (13, 15, 20, 21). One particular motif of the SPLUNC1 secondary structure that is 30 residues long, α4 (13), displays a helical structure (13, 18). We previously showed that the synthetic α4 region displayed modest antibiofilm property (22), which could be enhanced by optimization of the cationic amphipathic structure reminiscent of that of well-known natural AMPs (23–26). In this study, we sought to explore the potential of α4-derived antimicrobial peptides (α4-AMPs) to display broad-spectrum bactericidal and antibiofilm activities against the most common MDR pathogens. Considering that potential cytotoxicity and lack of *in vivo* efficacy are two major shortcomings of AMPs as potential therapeutics, we also determined the cytotoxicity of α4-AMPs to mammalian cells and *in vivo* efficacy in a respiratory infection model in mice.

## RESULTS

### Antimicrobial properties of synthetic α4 motif can be enhanced by sequence optimization.

**FIG 1:**
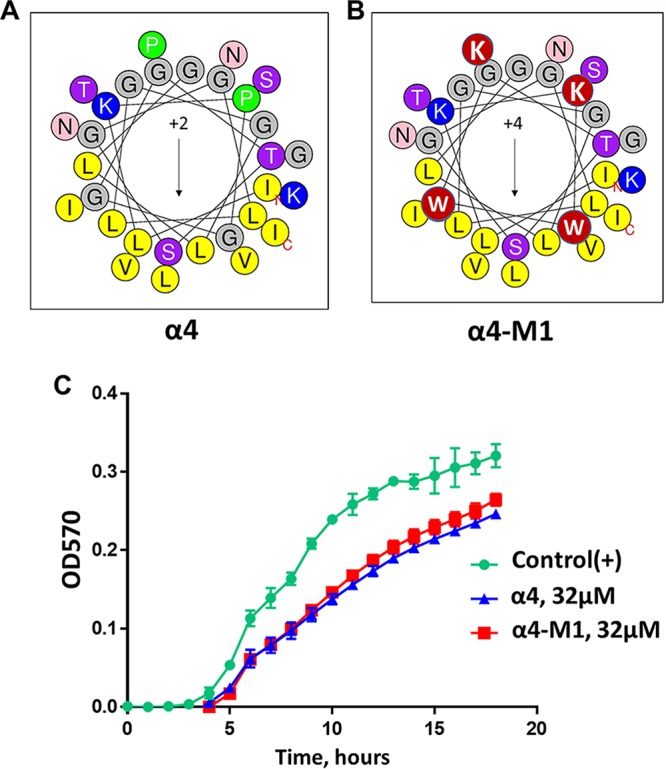
(A to C) Helical wheel diagrams (A and B) and activity of α4 and α4M1 against P. aeruginosa (C). In contrast to our previously published data on activity against S. aureus, the first-generation SPLUNC1-derived AMPs are ineffective against P. aeruginosa, as shown by the kinetics of growth inhibition, which is negligible compared to the untreated control. This is the rationale for further optimization. Red circles denote amino acid substitutions. OD570, optical density at 570 nm.

**FIG 2:**
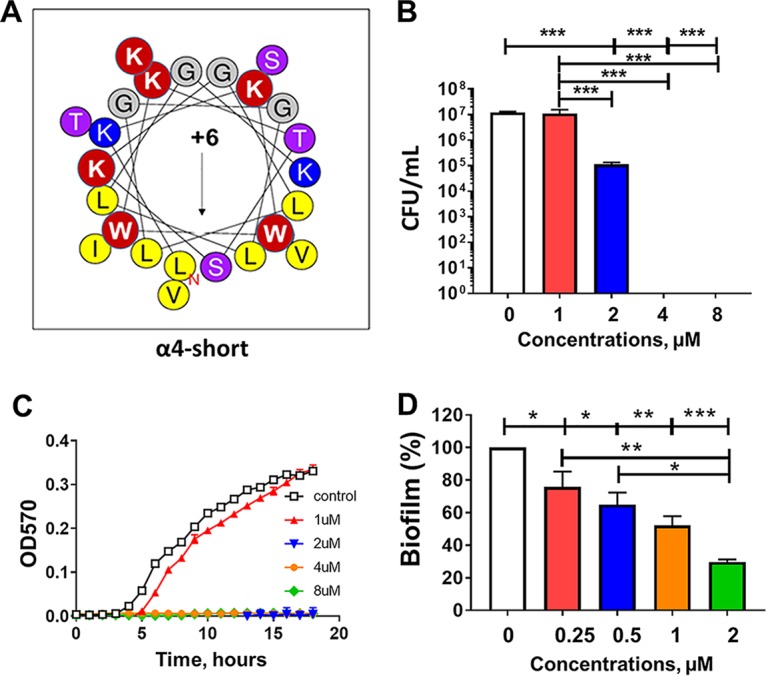
Helical wheel diagram and activity of α4-short against P. aeruginosa. (A) Helical wheel diagram. The black arrow indicates the direction of the hydrophobic moment, µH = 0.606 (http://heliquest.ipmc.cnrs.fr/), and red circles show the sites of amino acid substitutions (please refer to Table 1 for sequence alignment). In addition, unchanged hydrophobic and cationic residues are shown in yellow and blue, respectively. (B) Bacterial survival (PAO1) in the presence or absence of α4-short in cation-adjusted broth medium (MHB2) was determined as a function of peptide concentrations after 3 h of peptide treatment, using an initial inoculum of 10^6^ CFU/ml. (C and D) Kinetics of growth inhibition (C) and prevention of biofilm formation (D) were assessed as described in Materials and Methods. Note the significant effect on biofilm formation (D) at concentrations that are not effective against planktonic bacteria (B and C). Statistical significance was examined by Tukey’s multiple-comparison tests and indicated by bars and asterisks as follows; *, *P < *0.05; **, *P < *0.01; ***, *P < *0.001.

**FIG 3:**
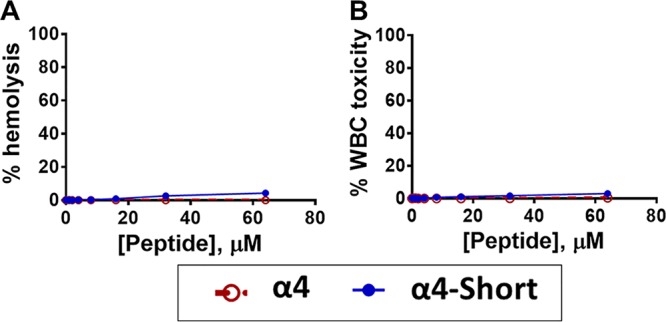
Negligible host toxicity of α4 and α4-short. (A and B) Freshly isolated human erythrocytes in PBS (A) or PBMC in DMEM (B) were incubated with each peptide at the indicated concentrations for 1 h, and the percentage of hemolysis or cytotoxicity was determined as described in Materials and Methods.

We previously demonstrated the antibiofilm activity of α4 and α4-M1 (both 30 residues long) against S. aureus, and these two AMPs displayed no toxicity to red blood cells (RBC) and white blood cells (WBC) (22). In this study, the goal was to determine whether these peptides (Fig. 1A and B) would demonstrate similar activity against P. aeruginosa and other ESKAPE pathogens. Surprisingly, the first-generation cationic peptides from SPLUNC1 were ineffective against P. aeruginosa at a concentration as high as 32 µM (kinetics of growth inhibition), as measured by optical density at 570 nm (OD_570_) (Fig. 1C). As a result, we aimed to broaden the scope of antibacterial properties of α4 by further enhancing the amphipathicity. Our approach was to reduce the length to 24 residues while increasing the number of Lys (positive charge) amino acids to six and leaving the number of hydrophobic residues unchanged (Fig. 2A), thereby increasing the density of the cationic and hydrophobic amino acids. These changes ultimately result in the new peptide named α4-short. The primary sequences and physicochemical properties (hydrophobic moment, hydrophobicity, and charge [Table 1]) and the helical wheel model represented in Fig. 2A indicate a progression toward a shorter length (24 residues) and more electropositive and amphipathic peptide, as measured by the hydrophobic moment (µH, 0.606), compared to either α4 or α4-M1 (27). We examined the novel peptide α4-short for its antimicrobial activity against P. aeruginosa (PAO1). The standard bactericidal assay using colony-forming unit (CFU) counting (Fig. 2B) demonstrated that the activity of the peptide is significantly enhanced compared to the parent peptide α4 and the first derivative α4-M1 (shown in Fig. 1), with a minimum bactericidal concentration (MBC_99_, 2-log-unit reduction in bacterial survival) of 2 µM in nutrient broth. A growth inhibition kinetic assay performed in nutrient broth shows no detected growth of P. aeruginosa at 2 µM (Fig. 2C). Further assessment of activity using the standard crystal violet biofilm detection method demonstrates a significant dose-dependent reduction in biofilm formed by P. aeruginosa (Fig. 2D). Of note, significant inhibition of biofilm occurred at concentrations that are not effective against planktonic bacteria (Fig. 2B and C). This finding suggests a mechanism similar to that of other AMPs previously investigated such as the engineered cationic peptide WLBU2 and the human AMP LL37 (2). As a primary characterization of the cytotoxic property, we compared the two peptides α4 and α4-short for hemolytic activity and white blood cell toxicity using freshly isolated human erythrocytes and peripheral blood mononuclear cells (PBMC). Both peptides show negligible hemolytic activity or WBC toxicity at concentrations up to 64 µM (Fig. 3). Thus, the structural modifications of α4 (α4-short) were sufficient to overcome the lack of activity of α4 against P. aeruginosa, in contrast to the amino acid substitutions resulting in the α4-M1 AMP. Notably, the optimized α4-short demonstrated a gain in antibacterial activity but did not cause significant increase in cytotoxicity, a common concern in AMP optimization.

**TABLE 1:** Changes in the primary sequence of α4 resulting in α4-M1 and α4-short

Peptide	Length (no. of amino acids)	Primary sequence^*a*^	Charge	μH^*b*^	H^*c*^	μH/H
α4	30	ILKPGGGTSGGLLGGLLGKVTSVIPGLNNI	2	0.373	0.558	0.668
α4-M1	30	ILK**KWW** GTSGGLLGGLLGKVTSVI**K** GLNNI	4	0.563	0.594	0.948
α4-short	24	LK**KWW** K TS K GLLGGLLGKVTSVI**K**	6	0.606	0.489	1.239

a Changes from α4 are shown in boldface type, and changes from α4-M1 are shown underlined.

b μH, hydrophobic moment.

c H, hydrophobicity.

### Antimicrobial activity of α4-short against MDR P. aeruginosa.

**FIG 4:**
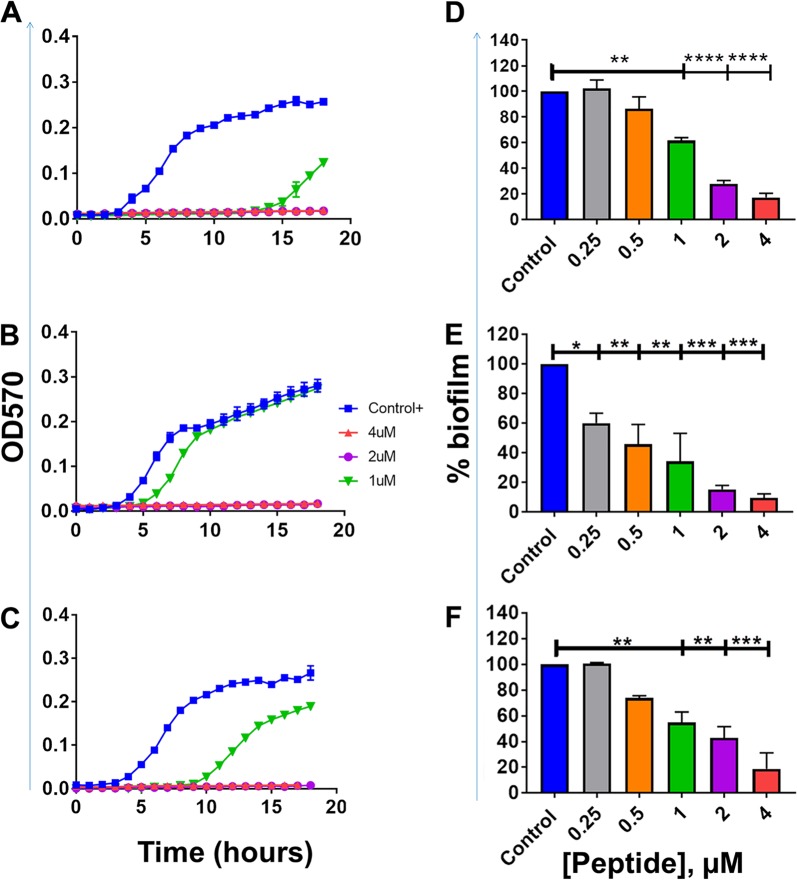
Activity against clinical strains of P. aeruginosa. (A to F) The peptide α4-short was examined for planktonic growth inhibition kinetics (A to C) and prevention of biofilm formation using the crystal violet detection method as described in Materials and Methods (D to F). (A and D) PA16-21; (B and E) PA474-1; (C and F) PA38-13. Statistical significance was examined by Tukey’s multiple-comparison tests and indicated by asterisks as follows: *, *P < *0.05; **, *P < *0.01; ***, *P < *0.001; ****, *P < *0.0001.

**FIG 5:**
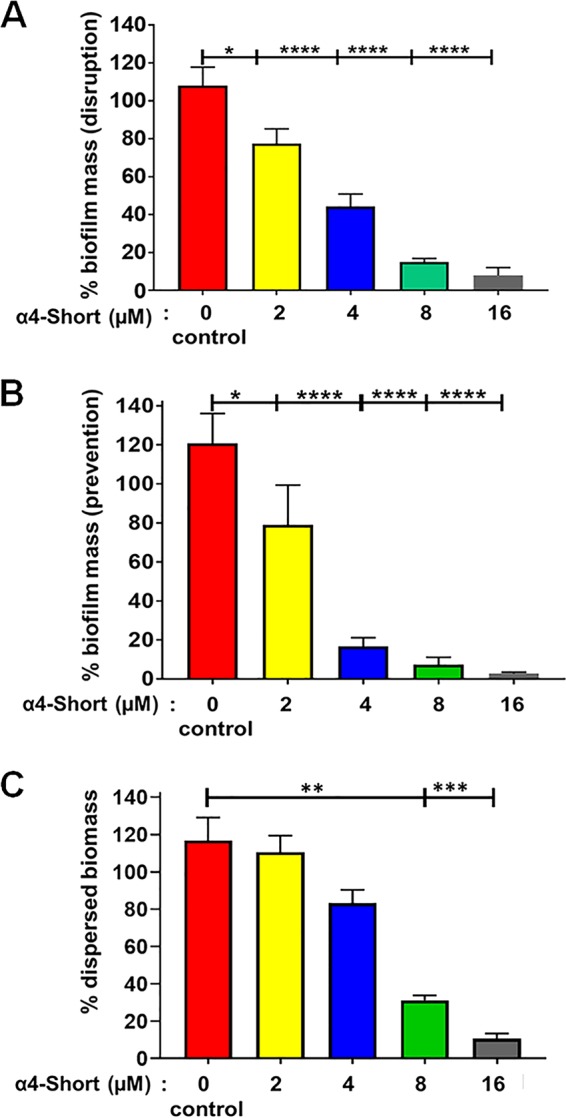
Disruption and prevention of P. aeruginosa-induced biofilm. (A and B) The peptide α4-short was examined for the ability to disrupt (A) or prevent (B) the formation of P. aeruginosa PAO1-induced biofilm using a biofilm-on-bead transfer model as described in Materials and Methods. (C) The prevention of dispersed bacteria in the form of planktonic and smaller biofilm pieces was also determined. Statistical significance was examined by Tukey’s multiple-comparison tests and indicated by asterisks as follows: *, *P < *0.05; **, *P < *0.01; ***, *P < *0.001; ****, *P < *0.0001.

**FIG 6:**
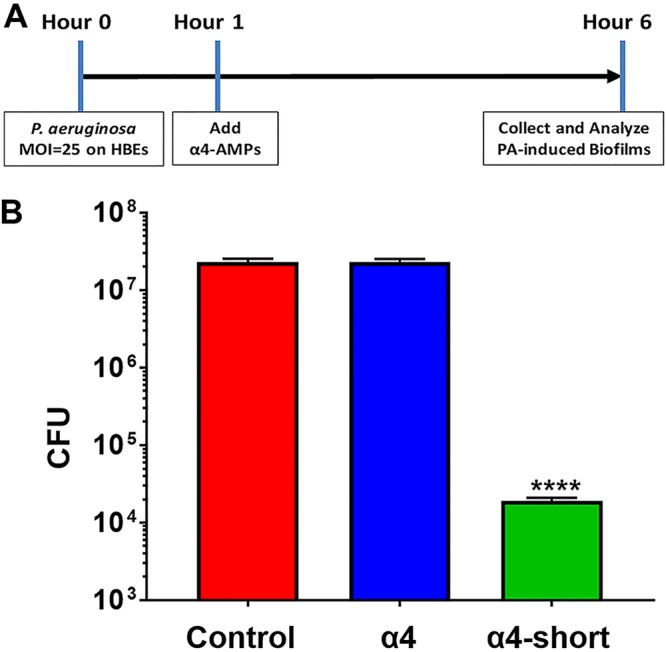
Prevention of P. aeruginosa-induced biofilm in a biotic coculture system. (A) Schematic timeline of the biotic coculture biofilm experiments. HBEs, human bronchial epithelial cells; AMPs, antimicrobial peptides; PA, P. aeruginosa. (B) The peptide α4-short was compared with the parent peptide α4 for the ability to prevent biofilm formation after attachment of P. aeruginosa (2 × 10^6^ inoculation/ALI insert) to polarized human airway epithelial cells at the apical side. Unattached cells were removed prior to addition of peptide. Five hours after the treatments, the sonicated biomass on epithelial cells was enumerated as CFU/milliliter. PBS was used as vehicle treatment control. Statistical significance was examined by Tukey’s multiple-comparison tests between α4-short and α4 or α4-short and the control indicated by asterisks as follows: ****, *P < *0.0001.

To examine whether or not the observed activity is strain specific, we further tested α4-short for bactericidal and antibiofilm prevention activities against three additional clinical MDR strains of P. aeruginosa (Fig. 4). The derived peptide α4-short displayed the ability to suppress the growth of all three MDR strains of P. aeruginosa at a concentration as low as 2 µM (Fig. 4A, B, and C). In addition, using the standard crystal violet method, we demonstrated that α4-short retained the ability to prevent biofilm formation against those strains with a remarkable reduction in the biofilm mass by 80 to 95% (Fig. 4D, E, and F) even at concentrations that are not effective against planktonic bacteria (concentrations as low as 0.25 to 1 µM). Due to the importance of biofilm in resistance to antibacterial treatment by standard antibiotics, we sought to further delineate the antibiofilm properties of α4-short using a biofilm-bead model, adapted from an evolutionary model as previously published (28). To execute this model, we allowed bacteria to grow in the biofilm mode on beads embedded in M63 overnight (Materials and Methods). It allows us to determine the effects of the peptide on biofilm disruption when peptide treatment occurs after the biofilm has formed on the bead. In addition, we were able to examine the degree of biofilm prevention activities, when a bead with preformed biofilm is transferred to fresh medium next to a sterile bead. Using P. aeruginosa (PAO1), we demonstrated a dose-dependent increase in the disruption of a preformed biofilm (Fig. 5A) with >95% biofilm disruption occurring at 16 µM. Similarly, α4-short prevents the migration of bacteria from one bead to the next bead, thereby effectively preventing new biofilm formation on sterile beads. Of note, the migration of bacteria from one bead to the next requires bacterial dispersion into the medium prior to attaching to the sterile bead. Again, consistent with data in Fig. 2 to 4, although α4-short was less effective (0 to 20% inhibition, *P* > 0.05) against dispersed (planktonic and aggregated biofilm breakout pieces) bacteria at lower concentrations (<4 µM), it remained significantly effective against biofilm formation (20 to 85% biofilm inhibition at <4 µM, *P* < 0.01). Finally, the antibiofilm property was further examined using our established biotic biofilm model that evaluated bacterial biofilm-forming activity on the surfaces of differentated airway epithelial cells (23, 29). After P. aeruginosa PAO1 was allowed to attach to polarized airway epithelial cells (AECs) on the apical side of air-liquid interface (ALI) cultured cells, the coculture was treated with either PBS (control), α4, or α4-short. Only cocultures treated with α4-short demonstrated a substantially lower level of biofilm formation (Fig. 6, *P* < 0.0001), with a 3-log-unit reduction of CFU counts at a modest concentration of 16 µM.

To more thoroughly examine the extent of the braod-spectrum activity of α4-short, we determined MICs in cation-adjusted nutrient broth (MHB2) against many MDR clinical strains of ESKAPE pathogens, including a large panel of MDR P. aeruginosa that were originally isolated from CF patients (3). As shown in Table 2, α4-short was effective against most common MDR strains at concentrations as low as 1 to 2 µM. Five strains required a MIC of 4 µM or 8 µM, while the highest MIC of 16 µM was observed against two of the 37 ESKAPE strains.

**TABLE 2:** Broad-spectrum activity of α4-short against clinical strains that are mostly MDR pathogens^*a*^

Strain	Activity^*b*^	MIC (µM)	
		α4-short	LL37
P. aeruginosa strains			
16-21	MDR	2	4
16-72	NKR	2	4
16-73	NKR	2	4
0038-13	MDR	2	2
0038-14	MDR	1	>16
0040-21	NKR	2	>16
0046-106	MDR	8	>16
0068-37	NKR	16	>16
0071-75	NKR	2	6
97-5	Bact-r	2	>16
109-10	Amik-r	2	6
132-37	NKR	2	>16
129-5	Lev-r	2	16
152-19	NKR	2	9
159-30	NKR	2	>16
474-1	MDR	2	4

S. aureus strains			
467-1	MRSA	2	>16
0154-17	MRSA	2	>16
187-10	MRSA	16	>16
0194-19	NKR	2	8
US300	MRSA	8	16
122-12	MRSA	2	>16

A. baumannii strains			
C3	NKR	1	1
D4	XDR	2	4
A2	XDR	4	2

E. coli strains			
YDC518	CRE	1	2
YDC575	CRE	2	8
YDC596	CRE	1	8
YDC107	CRE	1	2
YDC337	CRE	1	4
YDC748	CRE	2	2

E. faecium strains			
23614	VRE	1	8
14678	VRE	1	2
26125	VRE	1	8

K. pneumoniae strains			
A2	CRE	4	>16
D4	CRE	2	>16
438-16	NKR	4	>16

a Clinical strains of Pseudomonas aeruginosa (16 strains) and Staphylococcus aureus (6 strains) were from the Cystic Fibrosis Foundation. Clinical strains of Acinetobacter baumannii (3 strains), Escherichia coli (6 strains), Enterococcus faecium (3 strains), and Klebsiella pneumoniae (3 strains) were from the clinical laboratory of the University of Pittsburgh Medical Center via Yohei Doi’s laboratory.

b MDR, resistant to ≥3 classes of antibiotics; XDR, resistance to 10 classes of antibiotics; NKR, no known resistance; CRE, carbapenem-resistant *Enterobacteriaceae*; VRE, vancomycin-resistant *Enterococcus*; MRSA, methicillin-resistant *S. aureus*; Bact-r, bactrim resistant; Lev-r, levofloxacin resistant.

### The α4-short AMP demonstrated *in vivo* efficacy in a P. aeruginosa infection model.

**FIG 7:**
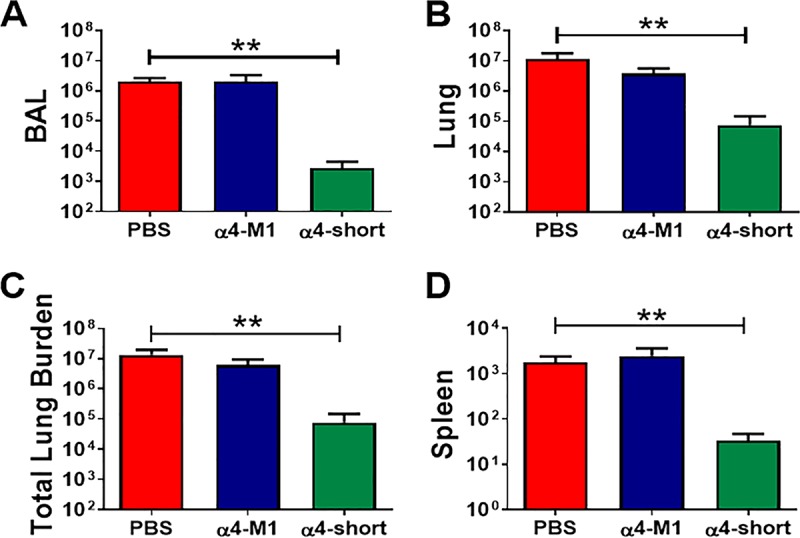
α4-short was effective at reducing bacterial burden in the mouse airway after exposure to P. aeruginosa. Bacterial burden was examined in CFU/ml (BAL [A]), CFU/g tissue (lung homogenate [B] or spleen homogenate [D]), or total CFU (BAL plus lung homogenate [C]) for mice treated with PBS (control), α4-M1, and α4-short. Peptides were administered at 0.05 mg/kg (1 µg) 1 h after bacterial exposure. Results are means plus SEM (error bars) from three independent experiments with six mice for each treatment group. One-way ANOVA was used to compare peptide-treated infected mice and PBS-treated infected animals. **, *P < *0.01, indicating statistically significant difference between control (PBS) and α4-short.

**FIG 8:**
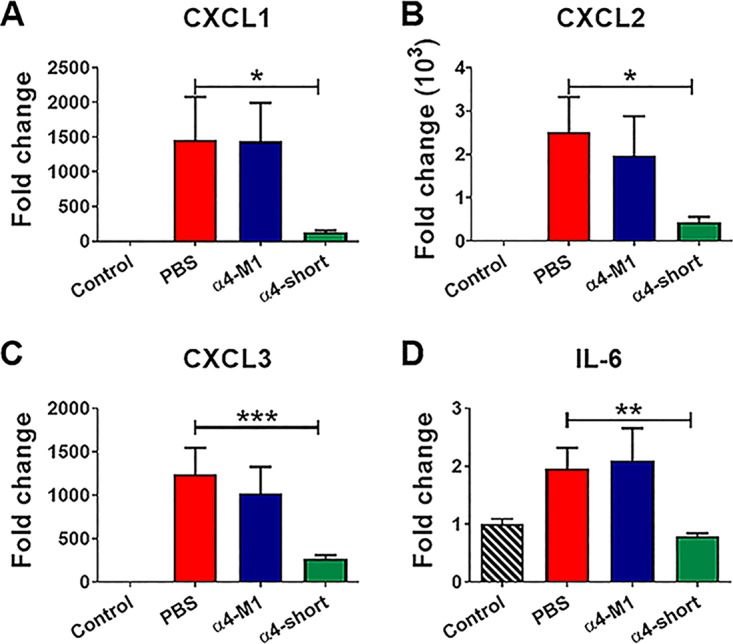
α4-short was effective at reducing inflammatory cytokines in mouse airways postexposure to P. aeruginosa. Inflammation-related cytokines (CXCL1 [A], CXCL2 [B], CXCL3 [C], and IL-6 [D]) in mouse lungs were examined by real-time qRT-PCR 24 h after peptides were administered at 0.05 mg/kg (1 µg) 1 h postinfection as shown in Fig. 7. Results are means plus SEM from three independent experiments with six mice for each treatment group. One-way ANOVA was used to compare peptide-treated infected mice and PBS (mock)-treated infected animals by the Kruskal-Wallis test. *Post hoc* comparisons were made using Dunnett’s multiple-comparison test. Values that are significantly different between PBS-treated (control) and α4-short-treated groups are indicated by asterisks as follows: *, *P < *0.05; **, *P < *0.01; ***, *P < *0.001.

In light of the antibiofilm activities of α4-short, particularly in bacterial coculture with AECs, we sought to examine the influence of α4-short on bacterial burden in a murine model of respiratory infection as proof of concept. Mice (C57BL/6; *N* = 6 for each group of every experiment) were infected intratracheally with P. aeruginosa PAO1. After 1 h, the infected animals were treated with AMPs or PBS also by intratracheal instillation (1 µg or 0.05 mg/kg of body weight), based on a small pilot experiment (data not shown) to determine minimum α4-short dosage protecting mice from a lethal infection at 1 h postexposure, as described in Materials and Methods. We monitored the animals for 17 h posttreatment (a total of 18 h postexposure) with no observed signs of morbidity (e.g., impaired mobility) in the α4-short-treated group. However, by 18 h postexposure, it became evident that the α4-M1- and PBS-treated mice were lethargic, progressing toward a moribund state. In contrast, the α4-short-treated mice appeared unaffected, with similar mobility to uninfected mice. On necropsies, mice treated with α4-short demonstrated signiﬁcantly (*P* < 0.001) reduced bacterial burden (CFU) by 1,000-fold in bronchoalveolar lavage (BAL) (Fig. 7A) fluid, and 100-fold in lung parenchyma (homogenate) (Fig. 7B), resulting in >100-fold reduction in total lung bacterial burden (Fig. 7C) compared to mice treated with α4-M1 and PBS (untreated control). A similar effect was observed in spleen homogenate (Fig. 7D). Further, we used quantitative real-time PCR to compare the levels of gene expression for inflammatory cytokines and chemokines. The levels of expression in α4-short-treated mice were markedly reduced compared to mRNA levels in PBS (mock)- and α4-M1-treated groups (Fig. 8A to C). In some cases, cytokine mRNA levels were reduced to baseline and similar to the uninfected mice (Fig. 8D). This substantial reduction in bacterial burden and inflammatory cytokines indicates the need to further explore the therapeutic potential of α4-derived AMPs.

## DISCUSSION

We report here the successful optimization of the primary sequence of the natural peptide α4, a specific helical region of SPLUNC1 (13, 15, 16, 18), based on the principle of cationic amphipathicity of helical AMPs (11, 23, 24, 30). Lessons learned from our previous studies of *de novo*-designed AMPs (e.g., Trp- and Arg-rich peptides) (3, 11, 30–32) indicate that increased host toxicity may be an off-target effect of AMPs that are optimized for antibacterial activity. While our *de novo* design inspired the structure-based optimization of α4 into the more potent and broadly active α4-short, compared to the parent peptide, the nontoxic property of the natural α4 motif represents an ideal template for sequence optimization with the potential to minimize host toxicity. Hence, we increased the density of cationic and hydrophobic residues by reducing the length of α4 from 30 to 24 residues, substituting two Trp residues, and tripling the positively charged residues using Lys. These moderate changes resulted in a newly designed peptide with broad-spectrum activity against both Gram-positive and -negative bacteria, including many clinical MDR strains of the ESKAPE pathogens. The activity also applies to antibiofilm properties, with the ability to both disrupt and prevent biofilm formation using the standard crystal violet or our bead transfer biofilm assay. Activity against biofilm, which may often be refractory to traditional treatment, is an early indication for potential application of α4-short to biofilm-related infections (e.g., airway and trauma-related infections). Indeed, the optimized peptide (α4-short) displayed negligible toxicity to mammalian cells, similar to its parent peptide (α4), providing the justified application to eradicate biofilm on differentiated AECs in a biotic biofilm system. Importantly, we concluded these initial studies with the demonstration of significantly enhanced *in vivo* efficacy, compared to α4, in a murine model of respiratory infection.

As the prevalence of MDR-related infections persists, it is urgent that we develop a new approach to antimicrobial therapy (33). The α4-derived AMP α4-short effectively inhibited the growth of all test MDR ESKAPE pathogens, with lowest activity against a selected group of 13 P. aeruginosa isolates and 4 MRSA isolates (3, 4, 29, 34–38). The thousands of AMPs recorded in multiple AMP databases and peer-reviewed data indicate that AMPs are highly diverse in secondary structures and display several antimicrobial mechanisms (39–43). However, we have focused primarily on the principle of helical amphipathicity (30, 44) because of the observation that membrane perturbation is predominant among cationic helical peptides, as it is the electrostatic interactions with the bacterial membrane that tend to induce the amphipathic helical structure with subsequent pore formation (45–51). Thus, structural optimization based on helicity is allowing us to exploit the concept of membrane perturbation as a way to overcome the most common resistance mechanisms exhibited by ESKAPE pathogens. Bacterial biofilm tends to display notably reduced susceptibility to antibiotics. In P. aeruginosa, for instance, a periplasmic glucan shield secreted by the bacteria tends to sequester antibiotics of different classes. This is particularly significant when an underlying pathophysiological aberration may facilitate chronic or recurrent infections, such as cystic fibrosis or other chronic illnesses. All the evidence thus far points to the ability of AMPs, including the naturally occurring ones, to overcome resistance despite being in settings that enhance the biofilm mode of growth. A plausible explanation is that the ability of AMPs to bind to the bacterial surface lipids may interfere with bacterial attachment to solid surfaces, an early step in biofilm formation. This model is consistent with the observation that α4-short is effective against biofilm at concentrations (<4 µM) that have minimal effect on planktonic bacteria in biofilm conditioned medium. In addition, these results (activity against both biofilm and planktonic bacteria) are supported by the observed differences between the well-studied AMPs WLBU2 and LL37 (2). Both α4-short and WLBU2 demonstrate that enhancing the cationic amphipathic motif markedly potentiates antibiofilm properties at low concentrations compared to LL37. Importantly, the primary sequence of the latter (14 different amino acids, 37 residues in length) is also more diverse in amino acid composition than that of α4-short (9 amino acids, 24 residues) or WLBU2 (only 3 amino acids, 24 residues). The diversity in amino acid composition suggests that several residues in LL37 may not be related to the determinants of antimicrobial function, which could explain the higher MIC of LL37 compared to the MICs of the engineered peptides. Importantly, the fact that the bactericidal activity of AMPs is indifferent to the metabolic state of the bacterial cells may partially overcome the biofilm mode of growth, as much as the peptide is able to reach the bacterial cells.

One of the most critical limitations of AMPs is the lack of evidence of efficacy in animal models. It is interesting that after 4 decades of AMP discoveries, there is still minimal *in vivo* data on AMPs. However, some topical applications in animal models in the last several years are particularly encouraging (52–56). Our group has been focusing on addressing this limitation, as demonstrated by the efficacy of engineered AMPs such as WLBU2 and natural AMPs, including frog skin-derived esculentin systemically and via direct delivery in the mouse airway (12, 23, 24, 31, 57, 58). Nevertheless, the increased host toxicity resulting from these optimization studies led us to adopt a natural template alternative, which facilitates the successful optimization of α4-short without the cytotoxicity observed in highly optimized *de novo*-engineered AMPs (22). The enhanced *in vivo* efficacy appears to be based on structural optimization of AMPs partly using Trp content. However, no guideline yet has been developed with respect to the positioning and density of Trp, although published data by the Vogel group suggest that Trp at the hydrophobic-hydrophilic interface of the amphipathic helix may be optimal (59). Further, the significance of this discovery, initiated by earlier studies of AMPs (e.g., HIV-1-derived AMPs, LLP1 and its derivative AMP WLSA5, and studies by Vogel and others [44, 51]) has not been fully examined. Such exploration is important for tailoring AMP development to the treatment of different types of infections and for enhancing the pharmacological properties such as peptide absorption, which would inform an ideal route of administration and bioavailability. Our study provides substantial evidence for the potential of a novel AMP to overcome the resistance property (based on numerous publications, including the most recent one [2]) that tends to be associated with biofilm formation, using complementary assays and models to test for activity and efficacy in airway-related infection by P. aeruginosa. While potential effects on tight junctions of HBE cells were not assessed because of the limitation to availability of the primary HBE cells, previous studies in our lab suggest that engineered AMPs with moderate toxic effects on RBC and WBC do not adversely affect transepithelial electrical resistance of HBE cells in ALI culture (23). Given the negligible toxicity of α4-short on the aforementioned cell types, it is predicted that the peptide would not affect barrier function.

The effectiveness of α4-short warrants the preclinical evaluation of α4-derived peptides to overcome biofilm-associated infections. However, despite these advances, it is critical to recognize that there are some important limitations to clinical development that must be overcome. There is still scant evidence for the absorption and bioavailability of AMPs, including the rare ones that display *in vivo* efficacy. It is anticipated that, despite *in vivo* efficacy in small-animal models, extensive efforts must be dedicated to enhancing the pharmacological properties of AMPs prior to clinical trials.

## MATERIALS AND METHODS

### Peptide synthesis.

Synthetic α4 (ILKPGGGTSGGLLGGLLGKVTSVIPGLNNI), α-4M1 (ILKKWWGTSGGLLG GLLGKVTSVIKGLNNI), and α4-short (LKKWWKTSKGLLGGLLGKVTSVIK) were synthesized using standard Fmoc (9-fluorenylmethoxy carbonyl) synthesis protocols as previously described (30), and purification was achieved by reversed-phase high-pressure liquid chromatography on a Vydac C_18_ or C_4_ column (The Separations Group). The identity of each peptide was established by MS (Electrospray Quatro II triple quadrupole mass spectrometer) (24).

### Bacteria.

PAO1 (ATCC BAA-47) is our P. aeruginosa laboratory strain, and all other bacterial strains are clinical isolates obtained from the cystic fibrosis (CF) core at Children’s Hospital in Seattle, WA, or anonymously provided by the medical laboratory of the University of Pittsburgh Medical Center. Bacteria were retrieved from −80°C freezer stock single colonies cultured on agar plates. Overnight bacterial cultures were diluted 1:20 with fresh cation-adjusted Mueller-Hinton Broth 2 (CAMHB2; Sigma) and cultured for an additional 2 to 3 h for exponential growth. Bacteria were centrifuged at 3,000 × *g* for 10 min. The pellet was resuspended in PBS (Sigma) to determine bacterial turbidity by optical density at 570 nm (OD_570_). Bacterial OD was always adjusted to 0.5 ± 0.01 (approximately 10^9^ CFU/ml) using a spectrophotometer to ensure reproducible results.

### Human airway epithelial cells.

Fully differentiated primary human airway epithelial cells (AECs) were derived from lungs removed at the time of lung transplantation at the Center for Organ Recovery and Education (Pittsburgh, PA, USA), as previously described (23). Epithelial cells were dissociated and seeded onto collagen-coated, semipermeable membrane inserts with a 0.4-μm pore size (Millicell-HA, surface area, 0.6 cm^2^; Millipore Corp., Bedford, MA). Cells were maintained in 2% Ultroser G medium at 37°C with 5% CO_2_. Twenty-four hours after seeding, the mucosal medium was removed, and the cells were allowed to grow at the air-liquid interface. Only well-differentiated cultures (>4 weeks old) were used in these studies.

### Biofilm assays.

**(i) Crystal violet method.** We used a slightly modified version of the microtiter plate assay as previously described (2, 15, 20, 60). Briefly, log-phase bacteria were diluted in DMEM (instead of rich broth to facilitate biofilm growth) to 10^8^ CFU/ml based on predetermined bacterial numbers that correlate with optical density using a spectrophotometer. A 50-µl volume of protein or peptide (in PBS), at different concentrations, was added to 50 μl of bacterial suspension in a sterile 96-well polystyrene plate. The final bacterial concentration of the mixture is 5 × 10^7^ CFU/ml, 50-fold higher than the 10^6^ CFU/ml value used in standard planktonic growth inhibition assays for adequate bacterial attachment, as required for biofilm formation. After 24 h of bacterial biofilm growth at 37°C (no shaking), the supernatant was discarded. The plate was washed with PBS prior to staining with 125 μl of 0.5% crystal violet (in 20% ethanol) for 15 min (15). Crystal violet-stained biomass was dissolved in 150 μl of 95% ethanol and measured using a plate reader at 620 nm.

**(ii) Bead transfer method.** We have developed a new biofilm assay using a bead transfer model which was previously used only for studies of bacterial evolution. Sterilized polystyrene beads (7-mm diameter) were submerged in DMEM with 5 × 10^7^ CFU/ml P. aeruginosa PAO1. The beads were incubated for 24 h at 37°C to allow maximum biofilm formation. The beads were then washed with PBS three times in order to remove any loosely attached planktonic bacteria. Each bead covered with PAO1 biofilm was transferred to a 2-ml microcentrifuge tube. To study the antibiofilm formation activity of antimicrobial agents, a new sterile bead was positioned on top of the PAO1 biofilm-containing bead. Peptides were then diluted to the desired concentrations in DMEM. A total of 1.5 ml peptide solution was added to the 2-ml microcentrifuge tubes to ensure that both beads were submerged in the treatment solution. The microcentrifuge tubes were then placed in a 37°C static incubator. Each treatment condition was done in triplicate with three sets of Eppendorf tubes containing two beads in each tube. After 18 h of treatment, all supernatant was removed from the tube, and the beads washed with PBS three times. Both beads in each tube were separately sonicated in 1.5 ml of PBS at 80% amplitude for 30 s using the DPS-20 dual processing system (PRO Scientific) (130 W) to dissociate the aggregated bacteria into single bacterial suspensions. Suspended bacteria were plated, and total CFU from each bead were assessed by CFU counting on tryptic soy agar plate. Compared to the positive control (DMEM without peptides), inhibition of biofilm formation from the top bead indicates biofilm prevention (biofilm migration) activity, while a reduction in biofilm mass from the bottom bead indicates biofilm disruption activity of the peptide (shown as a percentage).

**(iii) Biotic biofilm method.** Our biotic biofilm assay was performed using cocultures of polarized and well-differentiated human AECs and P. aeruginosa PAO1 with a starting multiplicity of infection (MOI) of 25 and 500 μl MEM on the basolateral side as previously described (15, 20, 23). Polarized human AECs were inoculated with P. aeruginosa PAO1 in 50 μl (initial inoculation ∼2 × 10^6^ per ALI culture insert) of MEM at the apical side for 1 h to allow attachment of the bacteria to the AECs in ALI culture. Unattached bacteria were removed prior to the addition of peptide. Each AMP (final concentration of 16 μM), diluted in 50 μl PBS, was added for 5 h. After a total of 6 h, the biofilms were disrupted by sonication for 30 s (PRO DPS-20 sonicator), with subsequent serial dilution and enumeration on tryptic soy agar plates to determine CFU.

### Bacterial growth inhibition assays.

Antibacterial activity was examined by a standard growth inhibition assay endorsed by the Clinical and Laboratory Standards Institute (CLSI) with minor modifications as follows (61). Bacteria were incubated with each of the indicated peptides in cation-adjusted Muller-Hinton broth (MHB2; Sigma-Aldrich) for 18 h, at which time *A*_570_ (absorbance at 570 nm) values were measured to examine growth inhibition using a BioTek microplate reader (BioTek Instruments) (11). MICs were defined as the peptide concentrations completely preventing detectable growth. Peptide concentrations of up to 16 µM were evaluated for antibacterial activity, with a starting inoculum of 10^6^ CFU/ml. To evaluate bactericidal activities, this assay was modified by treating bacteria with peptide for 3 h and enumerating the bacteria by serial dilutions and plating on agar at 37°C overnight (bacterial killing assay). MHB2 alone was used as negative control (vehicle) for all these assays (30).

### Red blood cell lysis assay.

Hemolytic assays were performed using red blood cells (RBCs) isolated from heparinized human blood samples from healthy donors (anonymously obtained from the Central Blood Bank of Pittsburgh) by Histopaque gradient centrifugation and then resuspended to 2.5% (vol/vol) in PBS as previously described (11). To determine RBC lysis, a volume of 50 μl (1:4) of the RBC suspension was mixed with peptides at variable concentrations ranging from 0 to 100 µM to a total volume of 200 µl in a round-bottom 96-well plate. The reaction mixture was incubated at 37°C for 60 min with gentle shaking. To analyze RBC lysis, the RBC-peptide mixture was spun at 600 × *g* for 5 min, and 80 µl of the supernatant was transferred to 120 μl (1:2.5) of RBC lysis buffer (final dilution, 1:10) in a flat-bottom 96-well plate for spectrophotometric analysis. Similarly, 0 to 50 µl of untreated RBCs was diluted in RBC lysis buffer to a final volume of 500 μl (up to 1:10 dilution), and the hemoglobin suspensions were used to produce a standard RBC lysis curve. The average absorbance values of the supernatants of all samples (200 μl) in triplicate were measured by using a microplate reader at 550 nm as an indicator of hemoglobin released from lysed cells. These experiments were verified by three independent trials.

### White blood cell toxicity assay.

We used flow cytometry as a direct measure of mammalian cytotoxicity to complement the hemolytic assays. Freshly isolated human PBMCs in RPMI plus 10% FBS were exposed to peptides or 70% ethanol (for 100% cell death) for 1 h at 37°C and immediately washed with PBS using a round-bottom 96-well plate. Fixable blue live/dead stain from Life Technologies was added to each sample according to the manufacturer’s instructions. The cells were washed with PBS and then fixed with 4 % formaldehyde (Thermo Scientific). After the cells were washed twice with PBS, the samples were stored at 4°C overnight prior to analysis by flow cytometry using a Guava flow cytometer (Millipore, MA, USA). Peptide-treated samples were compared with untreated control samples for reactivity with the blue dye, and data were analyzed using FLOWJO software (62). Percent toxicity was plotted in GraphPad (Prism software) against concentrations.

### Murine infection model.

The animal experiments were performed based on a protocol (protocol 17081148) approved by the Institutional Animal Care and Use Committee (IACUC) of the University of Pittsburgh based on the NIH guide for the care and use of laboratory animals. Seven-week-old female wild-type C57BL/6J mice were anesthetized by isoflurane inhalation and instilled intratracheally with ∼3 × 10^6^ CFU of P. aeruginosa PAO1 in 50 µl PBS. One hour after exposure, each peptide was intratracheally administered at 0.05 mg/kg (predetermined in a pilot experiment as the minimum dose of α4-short required to rescue mice from a lethal infection) in 50 µl of PBS. Control mice were given 50 µl of PBS without peptide. Mice were monitored for signs of morbidity and euthanized 18 h after bacterial exposure.

### Bronchoalveolar lavage and cytokine gene expression.

After 18 h postinfection, mice were euthanized for bronchoalveolar lavage (BAL) fluid and tissue isolation for mRNA expression. The trachea was cannulated to allow the lungs to be lavaged twice using 1 ml saline, and the BAL fluid and lung samples were collected in a biosafety cabinet (level 2). Bacterial cells were enumerated by serial dilution of BAL fluid and homogenized lung samples. For gene expression levels of different inflammation-related cytokines, total mRNA was isolated from the right lung using TRIzol reagent. Quantitative PCR was performed using ABI7900HT (Applied Biosystems, Foster City, CA, USA) and primers specific for the genes indicated in Fig. 8. Test and calibrator lung RNAs were reverse transcribed using a High-Capacity cDNA reverse transcription kit (Life Technologies). The cDNA was amplified as follows: 50°C for 2 min; 95°C for 10 min; 40 cycles, with 1 cycle consisting of 95°C for 15 s and 60°C for 1 min. Three replicates were used to calculate the average cycle threshold for each transcript of interest using β-glucuronidase for normalization (Assays on Demand; Applied Biosystems). Relative mRNA abundance was calculated using the ΔΔCt (cycle threshold) method.

### Statistical analysis.

The results are expressed as means ± SEM. Data normality was verified using both Shapiro-Wilk and D’Agostino and Pearson tests. For samples with normal distribution of the results, statistical comparisons between the groups of mice were made using analysis of variance (ANOVA), followed by Dunnett’s multiple-comparison test (one-way ANOVA). In some cases, unpaired *t* test was used for parametric statistical analysis. For the experimental results that did not pass the normality test, the Kruskal-Wallis test was used as the nonparametric alternative to the one-way ANOVA, and the Mann-Whitney test was used as the nonparametric alternative test to the independent sample *t* test. For *in vitro* experiments, statistical significance was examined by Tukey’s multiple-comparison tests. A *P* value of <0.05 was considered to be statistically significant.
